# Vascular emergencies—The new COVID‐19 crisis?

**DOI:** 10.1111/jocs.15072

**Published:** 2020-09-30

**Authors:** Pradeep Narayan, Gianni D. Angelini

**Affiliations:** ^1^ Rabindranath Tagore International Institute of Cardiac Sciences Kolkata India; ^2^ Bristol Heart Institute Bristol University Bristol UK

**Keywords:** acute limb ischemia, cardiac surgery, COVID‐19, DVT, vascular emergencies

While the main presentation and focus of the coronavirus disease 2019 (COVID‐19) has been lung injury, many other presentations have been reported since the start of the pandemic. The authors in this very pertinent and informative manuscript report the successful implementation of the “Hub‐and‐Spoke” model of healthcare delivery for vascular services in Lombardy, Italy during the early phase of the pandemic. More importantly, they have reported an increase in the number of vascular emergencies seen in this phase. The authors have further tried to explore if there is an association between this increase in vascular cases and the current COVID‐19 pandemic.^1^

The spectrum of vascular involvement experienced in the different “Hub” and “Spoke” hospital was varied. In the author′s own institution which was a “Spoke” hospital, the vascular presentation was mainly for aortic pathology. However, the “Hub” hospitals reported a significantly higher and unusual number of acute limb ischemia and amputations.^2^, ^3^ Seventeen cases of symptomatic carotid artery stenosis requiring carotid endarterectomy were also reported over a 7‐week period at another “Hub” hospital.^4^ Besides, an increase in the number of venous thrombosis and thromboembolism was reported as well.^5^, ^6^

This increase in the number of vascular cases, the majority of them requiring urgent attention, is a very interesting observation and deserves an in‐depth examination. Apart from assessing if this increase was driven by COVID‐19 we also must evaluate these cases for any differences in terms of presentations, pathogenesis, prognosis, and outcomes of operative interventions compared to non‐COVID‐19 patients.

While it is tempting to ascribe it to the “Hub and Spoke” model of service delivery for vascular emergencies and argue that the increase in limb ischemia was secondary to the concentration of vascular emergencies at the “Hub” hospitals it is quite likely that there is indeed “a vascular story” as the authors describe it, in COVID‐19 patients. When the number of cases reported at one of the “Hub” hospitals was compared with the preceding year it was seen that the increase was as high as nine times the volume reported during the same period in the preceding year. Similar increase was also seen in another “Hub” hospital which reported a sevenfold increase in the incidence of limb‐threatening ischemia.^4^ This kind of increase is unlikely to be the effect of the “Hub and Spoke model” alone. Moreover, this is not a phenomenon that is unique to Lombardy but is getting reported increasingly from other parts of the world too where different healthcare delivery models exist. There are several reports from other centers of young, nonatherosclerotic patients with COVID‐19 presenting with upper and bilateral lower limb ischemia as well as large‐vessel strokes.^7^, ^8^

It is also not the arterial system alone that seems to be affected by COVID‐19. In keeping with the author's observations, an increase in the prevalence of deep vein thrombosis (DVT) and venous‐thromboembolism among COVID‐19 patients has also been reported worldwide.^5^, ^6^ DVT has been reported to be as high as 46% and in the intensive therapy unit setting, is four times more common in patients with COVID‐19, compared to those without it.^9^, ^10^ Pooled data from 12 studies have reported the risk of venous thrombo‐embolism to be 38% in these cases despite prophylactic or therapeutic anticoagulation indicating a high risk of thromboprophylaxis failure.^11^ In a small early autopsy study in COVID‐19 deaths, unsuspected DVT was found in 58% of COVID‐19 patients, and pulmonary embolism was the cause of death in one‐third of the patients.^12^

In fact, as evidence grows it is now becoming apparent that the pathogenesis of organ dysfunction (lungs, kidneys, liver, and gastrointestinal system) in many of these patients was thrombotic in nature.^13^ And while the initial focus was mainly on microvascular thrombosis it now appears that there is a high incidence of macrovascular thrombosis as well as in COVID‐19. A simple and clinically relevant explanation for increased thrombogenicity has been provided using Virchow′s triad of hypercoagulability, stasis, and endothelial injury.^14^ The vascular endothelium is the cornerstone of organ dysfunction and endothelial dysfunction results in a prothrombotic state which can lead to the microthrombi formation as well as occlusion of bigger vessels.^15^ It has now been suggested that COVID‐19 is due to immune‐triggered, complement‐mediated microangiopathy.^13^

The presentation of vascular involvement in COVID‐19 as noted in the manuscript is extremely diverse. Apart from the aortoiliac thrombosis and involvement of both proximal and distal limb vessels involvement of the coronary arteries, subclavian artery, cerebral and carotid arteries, and mesenteric artery have all been reported. Similarly, apart from DVT, jugular and subclavian vein thrombosis, as well as prostatic plexus thrombosis, has been reported to occur in COVID‐19 patients.^6^, ^8^, ^12^, ^15^, ^16^, ^17^

What is especially worrying is the fact that limb ischemia is being reported in previously healthy patients with no comorbidities or history of peripheral vascular disease. Not only that, even after successful thrombo‐embolectomy and return of pedal pulses, recurrence of thrombosis within 2 h have been reported in the absence of atherosclerotic disease.^7^ Overall, the picture that emerges is that of a seriously deranged intravascular coagulation milieu and further illustrates that COVID‐19 is associated with previously underestimated but an inherently high risk of thrombogenicity.

Need for reintervention as well as lower than expected successful revascularization is another concern in the management of these patients.^2^, ^4^ The thrombus burden is significantly higher in these patients and there is a higher frequency of thromboses involving proximal vessels. Patients with symptoms of leg ischemia with concomitant COVID‐19 infection are more likely to require amputation. This association was found to be true even after adjustment for peripheral vascular disease. The likelyhood of death is also significantly higher in these patients. In presence of leg ischemia and COVID‐19 infection presence of pulmonary or systemic symptoms put them at higher risk of adverse outcomes.^16^

Thus, not only has the incidence of acute limb ischemia actually increases in COVID‐19 but the disease severity, prognosis, and outcome following surgical revascularization are also quite different when compared to patients with limb ischemia without concomitant COVID‐19.

While there is enough evidence available to suggest the presence of an increased association between COVID‐19‐infected patients and the risk of venous and arterial thrombosis the understanding of measures to improve outcomes is currently lacking. There is some suggestion that heparin usage may be associated with better outcomes as one of the studies showed that no patient who had received intravenous heparin required reintervention after revascularization. Even though this was a small study and statistically it was not a significant association it was suggested that the use of systemic heparin might improve surgical treatment efficacy, limb salvage, and overall survival. The benefit of heparin could be secondary to its anticoagulant effect as well as its anti‐inflammatory properties that include inhibitory interactions with multiple chemokines and complement.^15^, ^18^ Moreover, heparin might also have antiviral properties and prevents viral attachment by acting on the virus spike protein.^19^ Apart from anticoagulation, the influence of antiviral treatment, complement inhibition, immune‐suppression, plasma exchange, and intravenous immunoglobulins have to be evaluated in future studies.

The authors in this manuscript have initiated a very relevant discussion and have raised several important questions. Based on the evidence there is no doubt that the vascular burden in general and limb ischemia, in particular, is significantly increased by COVID‐19 infection. However, many unanswered questions remain, especially those pertaining to the management of this condition and improvement of outcome. Hopefully, future studies will help answer some of them.

## CONFLICT OF INTERESTS

The authors declare that there are no conflict of interests.
